# Assessment of a Rapid Semi-Quantitative Immunochromatographic Test for the Evaluation of Transfer of Passive Immunity in Calves

**DOI:** 10.3390/ani11061641

**Published:** 2021-06-01

**Authors:** Pauline Delhez, Elise Meurette, Emilie Knapp, Léonard Theron, Georges Daube, Anne-Sophie Rao

**Affiliations:** 1 RumeXperts, 4317 Faimes, Belgium; pdelhez@rumexpert.vet (P.D.); emeurette@rumexpert.vet (E.M.); leonardtheron@rumexpert.vet (L.T.); 2 Fundamental and Applied Research Center for Animal & Health (FARAH), Department of Food Sciences, Faculty of Veterinary Medicine, Food Microbiology, University of Liège, 4000 Liège, Belgium; Georges.Daube@uliege.be

**Keywords:** cattle, calf, immunoglobulin, passive immunity transfer, immunoassay, immunochromatography, refractometry, ELISA

## Abstract

**Simple Summary:**

Calves are born agammaglobulinemic and they rely on transfer of passive immunity (TPI) through ingestion of colostrum from the dam. Ensuring the effectiveness of TPI through blood serum immunoglobulins (IgG) quantification is of critical importance for the prevention of calf diseases. Therefore, this study primarily examined the performance of a novel on-farm quick test (SmartStrips^TM^, Bio-X Diagnostics, Rochefort, Belgium) to directly measure serum IgG concentration and assess TPI status in beef and dairy calves. Results showed that the quick test would provide veterinary practitioners and producers with an appropriate on-farm tool for direct calf serum IgG measurement and the assessment of TPI status. Such a tool would allow them, for instance, to take early actions regarding colostrum feeding practices or cow nutrition in the dry period to improve TPI and reduce calf morbidity, investigate calf health problems, or predict and measure the risk for pathology and antibiotic treatments in calves.

**Abstract:**

Calves are born agammaglobulinemic and they rely on transfer of passive immunity (TPI) through ingestion of colostrum from the dam. Ensuring the effectiveness of TPI through blood serum immunoglobulins (IgG) quantification is of critical importance for the prevention of calf diseases. The main objective of this study was to assess the performance of a novel on-farm immunochromatographic quick assay (SmartStrips^TM^, Bio-X Diagnostics, Rochefort, Belgium) compared to the ELISA reference method to directly measure serum IgG concentration and assess TPI status in beef and dairy calves. Additional comparison was made with the commonly used Brix refractometer. Jugular blood samples were collected from beef (*n* = 71) and dairy (*n* = 26) calves in Belgium within 7 days post-birth. Quantitative (Pearson correlation coefficients, Bland-Altman plots) and qualitative (diagnostic test characteristics, weighted kappa for classification into 4 categories of TPI) analyses were performed to evaluate the performances of the quick test and the refractometer compared to ELISA. The quick test showed a correlation of 0.83 and a classification agreement (weighted kappa) of 0.79 with the reference method (average values for two types of blood anticoagulants). Performances were better for low IgG concentrations and the assessment of poor TPI status and they outperformed those of the Brix refractometer. Results suggested that the immunochromatographic quick test can be considered as a suitable on-farm method for direct serum IgG measurement and the assessment of TPI status in calves, contributing to timely interventions in the management of calves with inadequate TPI.

## 1. Introduction

Calves are born agammaglobulinemic as the structure of the bovine placenta prevents the transfer of immunoglobulins (Ig) from the dam to the fetus [1]. Thus, calves are dependent on the absorption of Ig from colostrum, the initial secretion from the mammary gland after parturition, for the transfer of immunity from the dam before their own immune system becomes active [2]. Immunoglobulins are absorbed into the calf’s blood through the intestine and this absorption ceases approximately 24 h after birth due to the rapid modification of the gut enterocytes, meaning that early administration of colostrum is essential [3]. This transfer of Ig from the dam colostrum to the offspring is referred to as “passive transfer of immunity” or “transfer of passive immunity” (TPI) [4]. The most predominant Ig in bovine colostrum and serum is immunoglobulin G (IgG) [5]. Consequently, TPI is assessed with reference to the concentration of this specific Ig [6]. The passive immunization is essential for the calf to survive environmental pathogen challenges in the first weeks of life until it is able to synthesize its own antibodies, i.e., from around 2 to 4 weeks of age [2,7]. Failure of TPI occurs when calves have too low IgG concentrations in their blood measured between 24 h and 7 days after their birth. The standard for failure of TPI was generally defined as IgG concentration in the serum <10 g/L [8,9,10], but more recently a group of calf experts proposed a new standard including four categories—excellent, good, fair, and poor TPI—with serum IgG levels of ≥25.0, 18.0–24.9, 10.0–17.9, and <10 g/L, respectively [4]. Failure of TPI depends on several factors, the most important being poor colostrum quality (i.e., low concentration of IgG), insufficient volume of colostrum administrated, delay of colostrum feeding, or inadequate intestinal development of calves [2,8,11]. Many studies have reported that failure of TPI is associated with higher risk for illness and death in the young age as well as reduced growth rate [12,13,14,15]. Evaluating the adequacy of TPI in neonatal calves is therefore of great importance, allowing interventions and investigations into colostrum management and calf health problems. TPI can be quantified by measuring calf blood IgG during the first week of life using direct or indirect immunologic and biochemical methods [10,16,17,18]. Direct reference laboratory methods to measure IgG concentration include the radial immunodiffusion assay (RID) and the enzyme-linked immunosorbent assay (ELISA) [16,19,20,21]. However, these methods are time-consuming, technically demanding and expensive [22,23,24]. So, a number of on-site rapid methods have been developed [25,26,27]; a common one being the Brix refractometer that provides measurement of total solids which correlates to total protein concentration in the serum. Because Ig account for a large proportion of the proteins in the serum of newborn calf and non-Ig protein proportion is almost constant, Brix refractometers can provide an estimate of serum IgG concentration [10,28,29]. More recently, a new rapid on-site test based on the immunochromatography method has become commercially available (SmartStrips™, Bio-X Diagnostics, Rochefort, Belgium). Immunochromatography is a technique in which immunochemical reactions are carried out on a chromatographic paper by capillary action [30]. This novel semi-quantitative test provides a value of the IgG concentration in calf blood serum within 15 min. Depending on the level of reliability of the test, it could be easily used by veterinarians for rapid monitoring of TPI in dairy and beef calves. As such, the main objective of this study was to assess the performance of the immunochromatographic quick test compared to the ELISA reference method to quantify serum IgG concentration and assess the TPI status in dairy and beef calves. Secondary objectives were: (1) a comparison with the commonly used digital Brix refractometer, and (2) the evaluation of the effect of the anticoagulant type (ethylene-diamine-tetra-acetic acid (EDTA) vs. lithium heparin, which are common anticoagulants) in the blood sampling tubes on the performances of the quick test.

## 2. Materials and Methods

### 2.1. Animals and Sample Collection

A total of 26 clinically healthy dairy calves (25 purebred Holstein and one purebred Jersey—eight males and 18 females) and 71 clinically healthy beef calves (purebred Belgian Blue—39 males and 32 females) from 21 commercial herds in Belgium were sampled in October and November 2020. We estimated the number of calves needed for this study based on failure of TPI (i.e., poor TPI) in the reference population ranging from 25% [31] and 35% [32]. We used the WinEpi software (www.winepi.net, accessed on 1 October 2020) to estimate the sample size, assuming a reference population size of 1 million calf births per year in Belgium, a confidence level of 95% and an accepted error of 10%. The estimated minimum sample size was 88 calves for a prevalence of failure of TPI of 35% and 73 calves for a prevalence of 25%. Health status was based on a physical examination. Clinically healthy calves did not show any clinical signs of past or present disease (e.g., no sign of diarrhea or respiratory disease, no dehydration, adequate appetite, adequate responsiveness) and had a rectal temperature <39.5 °C. Calves were administered between 1.5 and 8 L of colostrum within 12 h of birth by nipple bottle or drench probe (i.e., there was a wide variability in colostrum amount, time of administration, and systems of administration). Blood sampling was performed by veterinarians as part of the regular veterinary monitoring on farms. Sample selection was based on the following criterions: voluntary participation of farmers as part of the regular veterinary monitoring in routine during the reporting period, age of calves (from 2 to 7 days old), clinically healthy status, breed, and diversity in colostrum feeding practices (to have enough variability in serum IgG concentrations). Newborn calves IgG testing (using Brix refractometry) was systematically used as a normal practice before the beginning of the trial in all the farms enrolled in the study, and the test was performed not only on the calves used for the study but also on the other calves of the farms. 

Blood was collected from the 2 to 7-days-old calves by jugular venipuncture, using a 20-gauge, 1.5-inch hypodermic needle (BD Vacutainer^®^ PrecisionGlide^TM^ Multiple Sample Needle, Becton, Dickinson and Company, Franklin Lakes, NJ, USA), into different tubes: (1) a 8.5 mL sterile, plastic Vacutainer tube with an inert gel and without anticoagulant (BD Vacutainer^®^ SSTTM II Advance); (2) a 10 mL sterile, plastic Vacutainer tube with ethylenediamine tetraacetic acid (EDTA) anticoagulant (BD Vacutainer^®^ K2E (EDTA)); and (3) a 4 mL sterile, plastic Vacutainer tube with lithium heparin anticoagulant (BD Vacutainer^®^ LH 68 I.U.). The samples in the tubes without anticoagulant were allowed to clot, and serum was harvested within 4 h of collection by centrifugation at 2900× *g* for 15 min at ~17 °C. One aliquot of serum was used immediately after centrifugation to determine the percentage of total solids in serum (digital Brix refractometer). Another aliquot was stored in one micro-tube (1.5 mL) and then frozen at −20 °C before IgG concentration determination using ELISA (considered as the reference standard). The tubes with anticoagulant (EDTA or lithium heparin) were stored at 4 °C for maximum 24 h before IgG testing using the immunochromatographic assay kits.

### 2.2. ELISA (Reference Method)

A commercially available ELISA kit (BIO K 165-QuantELISA Bovine Immunoglobulin/competition; Bio-X Diagnostics) was used to provide a reference standard of IgG concentration in the blood serum. RID was normally considered as gold-standard for IgG quantification, but previous studies showed that IgG concentrations determined by ELISA and RID were highly correlated [6,16,33]. The principle of the assay is to identify the presence and to measure the concentration of serum IgG using microplates sensitized with protein G specific for immunoglobulins. The ELISA kit was used according to the manufacturer’s recommendations. In brief, the serum samples in the dedicated micro-tubes were allowed to thaw at room temperature before analysis. Colostrum calibrator powder was diluted using phosphate buffered saline (PBS) to produce 8 IgG standard solutions (166,666, 111,111, 74,074, 49,383, 32,922, 21,948, 14,632, and 9754 ng of IgG/mL) for the calibration curve. The calf serum samples were diluted 1/100 in PBS. The standard solutions and all samples were added to a dilution microplate (100 μL/well), in duplicate. The horseradish peroxidase conjugate was diluted 50-fold in the dilution buffer (i.e., 250 μL of conjugate in 12.25 mL of dilution buffer) and 100 μL of this solution was added to each well of the microplate. A volume of 100 µL of the dilution plate was transferred to a 96-well test microplate sensitized with protein G specifically against IgG. The microplate was incubated at room temperature for one hour. The test microplate was washed three times using a washing solution. A volume of 100 µL of chromogen solution was added to each well and the plate was incubated at room temperature and away from light for 10 min. Stop solution (50 µL) was added to each well. The optical densities were recorded using a microplate spectrophotometer with a 450 nm filter. A standard calibration curve was constructed using a four-parameter curve fit using the average values from duplicate standard wells. This curve represented the relationship between optical densities (absorbance) and IgG concentration. The values of the curve were interpolated to obtain the IgG concentration of the calf serum samples. In the end, the obtained IgG concentrations were corrected with a 1.12 factor so that values were comparable with RID following recommendations of the manufacturer and the literature (e.g., [6]).

### 2.3. Brix Refractometry

The blood serum aliquots were tested using a digital Brix refractometer (Milwaukee MA871 Refractometer, Milwaukee Instruments, Rocky Mount, NC, USA). The refractometer determines the Brix score of serum by shining light through the sample, measuring the refractive index of serum, and presenting the reading in Brix units on a digital scale from 0 to 85%. The refractive index estimates the total solids concentration in the serum, which is an indication of the IgG concentration. The Brix refractometer was used according to the manufacturer’s recommendations. In brief, the sample was dripped onto the prism surface of the refractometer using a pipette until the well was completely filled. The measurement was displayed in units of % Brix. After the measurement, the sample was removed from the well by absorbing with a soft tissue. The prism and well were rinsed using distilled water and wiped dry. The refractometer was calibrated with distilled water before each day of use.

### 2.4. Immunochromatographic Assay Kits (SmartStrips™)

**Figure 1:**
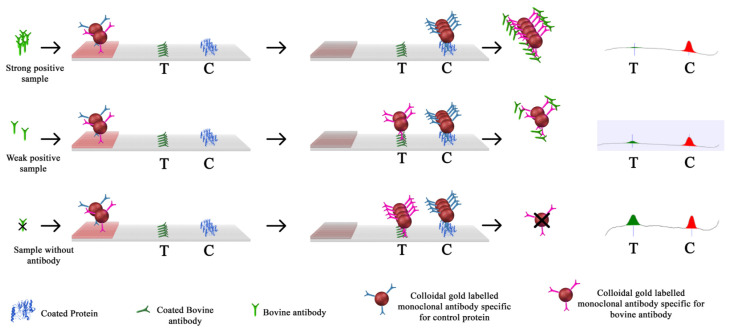
Schematic representation of the quick test (SmartStrips^TM^, Bio-X Diagnostics, Rochefort, Belgium) mechanism to estimate calf serum IgG concentration. T = test. C = control.

The blood samples with anticoagulant (EDTA or lithium heparin) were tested with an immunochromatographic assay kit (SmartStrips™, Bio-X Diagnostics). The immunochromatographic assays are run on whole blood for greater practical convenience (i.e., no need to wait and centrifuge to obtain serum), but results are expressed in serum IgG concentrations (mg/mL). The test principle is illustrated in Figure 1. The test uses two visual lines, a test line and a control line, to provide a semi-quantitative determination of the concentration of bovine serum IgG in blood samples. The blood sample first encounters mobile (i.e., unbound) antibodies specific to bovine IgG labelled with colloidal gold particles and mobile antibodies specific for control proteins. Then the sample encounters the test line with bovine IgG coated on the surface. The test is based on the competition between the bovine IgG in the sample and the bovine IgG coated on the test line for the gold labeled mobile antibodies specific to bovine IgG. When IgG are absent from the blood sample, mobile antibodies will bind to the coated IgG on the test line and a visual marker will show. Conversely, when IgG are present in the blood sample, they bind to the gold labeled mobile antibodies to prevent them binding to the fixed IgG on the test line, and thus no visual marker shows. Gold labelled antibodies specific for control proteins bind on the control line to confirm that the test has operated correctly. The higher the concentration of IgG in the blood, the stronger the competition for the gold particles of the corresponding mobile antibodies, in such a way that these mobile antibodies do not accumulate on the test line. The intensity of the test line is inversely proportional to the concentration of IgG in the blood. The assay results are read with a smartphone camera using the SmartStrips^TM^ App application and are interpreted to obtain the serum IgG concentration. Serum IgG concentration values are provided between 2 and 25 mg/mL. Values lower than 2 mg/mL are displayed as <2 and values higher than 25 are displayed as >25. The resulting data can be exported to a remote computer or server.

The quick tests were used according to the manufacturer’s recommendations. In short, blood samples with the EDTA or lithium heparin anticoagulant were allowed to warm up to room temperature. For each sample, after homogenization, 10 µL of blood was taken up using a mini pipette and diluted in the eluent provided in the test kit. The solution was homogenized and the SmartStrips^TM^ assay was placed immediately in the liquid. After waiting for 10 + −1 min, the results (serum IgG concentration in mg/mL) were read under appropriate light conditions using a smartphone with the SmartStrips^TM^ App application. The manufacturer did not provide any data about the repeatability and reproducibility of the test.

### 2.5. Statistical Analyses

Statistical analyses were carried out using the software R (version 4.0.3 [34]) and Microsoft Excel 2016. Regarding the quick test, samples with IgG values indicated as <2 and >25 mg/mL had to be excluded from the quantitative analyses (leaving 91 samples for EDTA and 87 samples for lithium heparin), but they were kept for qualitative analyses.

#### 2.5.1. Quantitative Analyses

Descriptive statistics were calculated for IgG values of ELISA and the two SmartStrip^TM^ tests (with EDTA or lithium heparin used as anticoagulant in the sampling tube) as well as for Brix scores of the digital refractometer.

The following distributions were plotted: (1) IgG measured by the quick test (EDTA or lithium heparin, respectively) vs. IgG measured by ELISA (*n* = 91 and 87, respectively), (2) Brix scores vs. IgG measured by ELISA (*n* = 97), and (3) IgG measured by the quick test with EDTA vs. IgG measured by the quick test with lithium heparin (*n* = 86). From these plots, Pearson’s correlation coefficients were calculated. 

Bland-Altman plots were used to assess: (1) the agreement between the reference method (ELISA) vs. the quick test (with EDTA or lithium heparin) and (2) the agreement between the quick test with EDTA vs. with lithium heparin. The Bland-Altman plot depicts the differences between the measurements of the two methods of interest which are plotted against the average measurements of these two methods [35]. To be meaningful, the two methods need to be on the same scale (i.e., Brix refractometry could not be assessed with Bland-Altman plots). Horizontal lines are drawn at the mean difference (i.e., representing the mean bias) and at the limits of agreement, which are defined as the mean difference +/− 1.96 times the standard deviation of the differences. The limits of agreement should be discussed in light of the clinical relevance. Two series agree when one does not over- or underestimate the other excessively (i.e., no excessive bias), and if differences between the two measurements for each individual are not too important [35].

#### 2.5.2. Qualitative Analyses

Four classes were defined to categorize the level of TPI in calves. These categories were based on the new standard classification defined in the United States by Lombard et al. [4], adapted for Belgium following advice of field experts in animal health (i.e., the limit for poor TPI was still fixed at <10 mg/mL, but the ranges of IgG concentrations of the categories of fair and good TPI were more narrow and excellent TPI was set at IgG concentration of ≥20 mg/mL). These categories are described in Table 1 with limits expressed in IgG (mg/mL) and in equivalent Brix measurement (Brix %). The equivalent Brix % cut-off points were defined based on multiple studies [4,28,36].

Diagnostic test characteristics (using ELISA as reference method) were determined to assess the effectiveness of the quick test (with EDTA or lithium heparin as blood anticoagulant) at correctly classifying calves into the four categories of TPI status. Comparison was made with test characteristic performances of the digital Brix refractometer. Common test characteristics relevant for multi-class diagnosis are precision (also called positive predicted value), sensitivity (also called recall), and F1 score that were calculated for each of the 4 TPI categories. The macro F1 score and the overall accuracy were also calculated. Precision measures the proportion of positive identifications (i.e., predictions attributed to the considered category) that were correctly identified. Sensitivity measures the proportion of actual positives (i.e., samples belonging to the considered category as determined by the reference method) that were correctly identified. F1 score is defined as the harmonic mean of the precision and sensitivity [37]. Macro F1 score represents the average of F1 scores of each category. Overall accuracy is defined as the proportion of samples that were correctly classified by the test among all the categories.

Weighted kappa coefficient was calculated to assess the agreement between the different tests. It calculates the agreement between measurements of multiple-level categorical variables taking into account the expected agreement by chance and the closeness of agreement between categories [38]. All levels of disagreement between methods were weighted equally (linear weights). Positive weighted kappa values can be interpreted somewhat arbitrarily as follows: <0.2 = poor agreement, 0.21–0.40 = fair agreement, 0.41–0.60 = moderate agreement, 0.61–0.80 = good agreement, 0.81–0.99 = excellent agreement, and 1 = perfect agreement [17,38].

## 3. Results

### 3.1. Quantitative Analyses

#### 3.1.1. Descriptive Analysis

Descriptive statistics for the measurements of the different methods are shown in Table 2. Mean IgG concentration measured by ELISA was 13.6 (SD = 7.5), with a range from 1.3 to 37.1 mg/mL. Mean Brix refractometer value was 8.4% (SD = 0.9), with a range from 6.6 to 10.3. Mean IgG concentration measured by the quick test was 12.8 and 12.0 (SD = 5.7 and 5.3) for EDTA and lithium heparin anticoagulants, respectively. Minimum and maximum values for the quick test were not inferior to 2 and did not exceed 25, as conditioned by the lower and upper limits of the method.

#### 3.1.2. Correlation Coefficients

**Figure 2:**
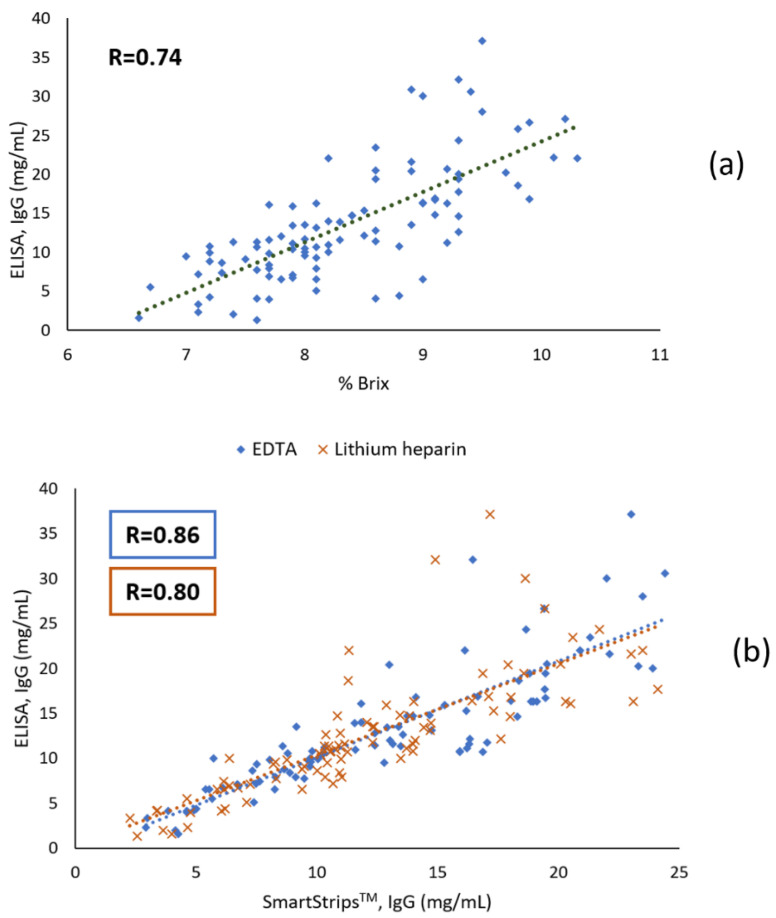

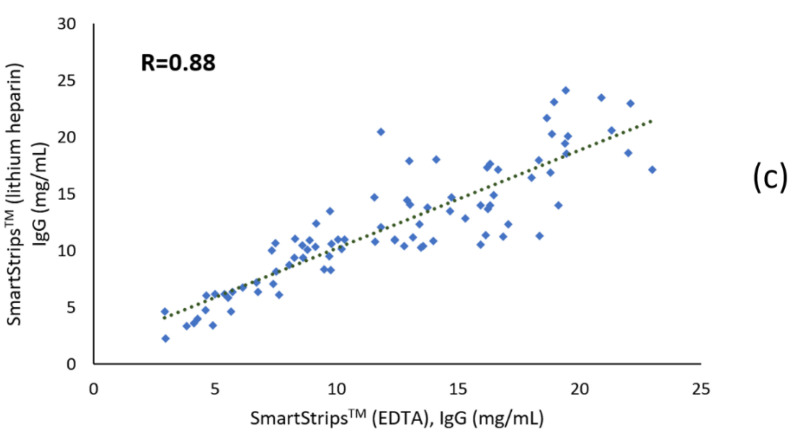
(**a**) Serum IgG concentration determined by ELISA, compared with %Brix of calf serum (*n* = 97); (**b**) Serum IgG concentration determined by ELISA, compared with serum IgG concentration determined by the quick test (SmartStrips^TM^) with EDTA or lithium heparin anticoagulants (*n* = 91 and 87, respectively) (**c**) Serum IgG concentration determined by the SmartStrips^TM^ with lithium heparin anticoagulant, compared with serum IgG concentration determined by the SmartStrips^TM^ with EDTA anticoagulant (*n* = 86).

Figure 2 shows scatter plots and Pearson correlation coefficients for (1) the Brix refractometer vs. ELISA, (2) the quick test (EDTA or lithium heparin) vs. ELISA, and (3) the quick test with EDTA vs. quick test with lithium heparin. Results from the quick test were more closely correlated to ELISA (r = 0.86, *p* < 0.01 and 0.80, *p* < 0.01 for EDTA and lithium heparin anticoagulants, respectively) than results from the Brix refractometer (r = 0.74, *p* < 0.01). Results from the quick test with EDTA vs. heparin lithium were highly correlated (r = 0.88, *p* < 0.01).

#### 3.1.3. Bland-Altman Plots (Agreement)

**Figure 3:**
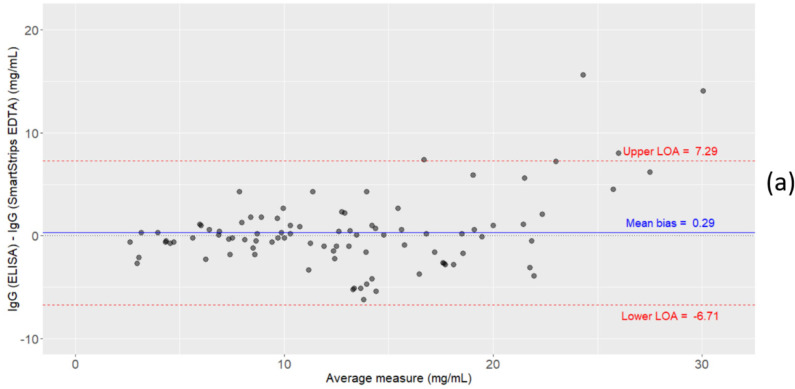

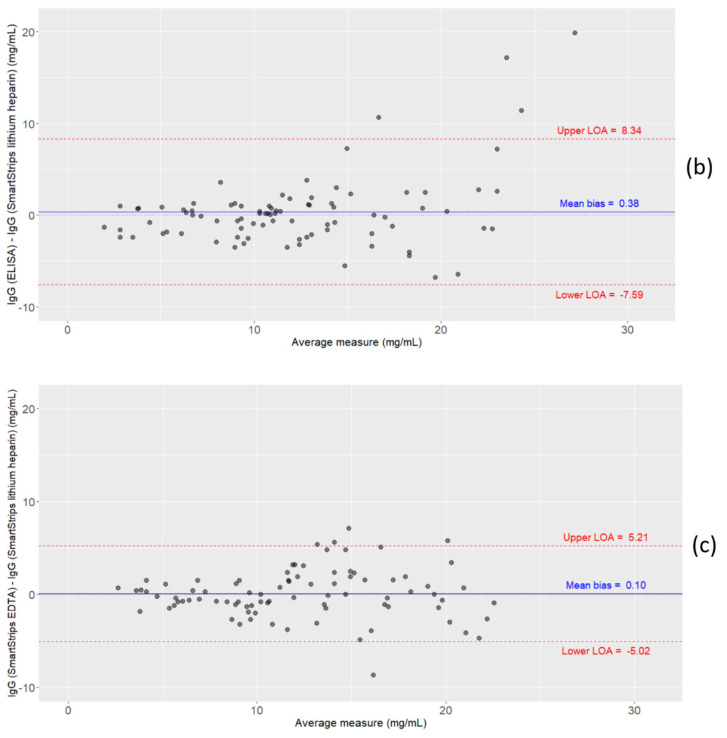
Bland-Altman plots showing the level of agreement between serum IgG concentration (mg/mL) measured by (**a**) ELISA vs. the quick test (SmartStrips^TM^) with EDTA (*n* = 91), (**b**) ELISA vs. SmartStrips^TM^ with lithium heparin (*n* = 87), and (**c**) SmartStrips^TM^ with EDTA vs. SmartStrips^TM^ with lithium heparin (*n* = 86). LOA = limit of agreement.

The Bland-Altman plots comparing the measurement of serum IgG concentrations using: (1) ELISA vs. quick test with EDTA, (2) ELISA vs. quick test with lithium heparin, and (3) quick test with EDTA vs. quick test with lithium heparin, are shown in Figure 3. There was a negligible mean bias for the quick test measurements compared to ELISA measurements (0.29 and 0.38 for EDTA and lithium heparin, respectively). The limits of agreement were −6.71 and 7.29 for the quick test with EDTA anticoagulant, and −7.59 and 8.34 for the quick test with lithium heparin anticoagulant compared to ELISA. There was a close-to-zero mean difference between the IgG measurements using the quick test with EDTA and the quick test with lithium heparin, with limits of agreement of −5.02 and 5.21.

### 3.2. Qualitative Analyses

#### 3.2.1. Descriptive Analysis

**Figure 4:**
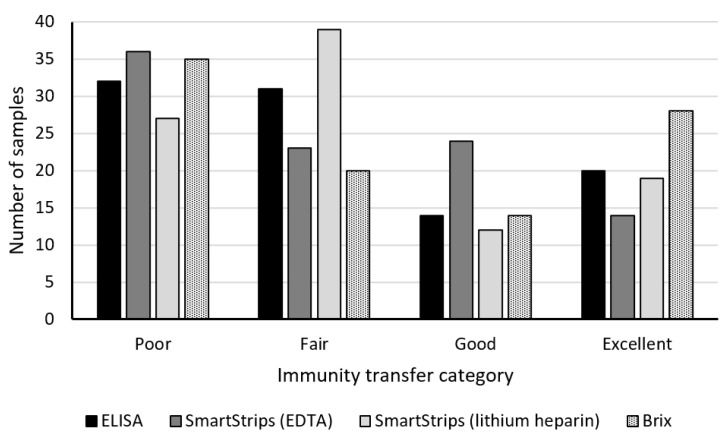
Sample size for each category of TPI status for ELISA, the quick test (SmartStrips^TM^) with EDTA anticoagulant, SmartStrips^TM^ with lithium heparin anticoagulant, and Brix refractometer (total *n* = 97 for each method). See Table 1 for the definition of the categories.

The sample size for each category of TPI status is shown in Figure 4. The number of samples per category ranged from 12 to 39. The category of poor TPI had the largest number of samples for all methods, except for the quick test with lithium heparin anticoagulant. The category of good TPI was the smallest for all methods except for the quick test with EDTA anticoagulant. We observed that 33% of calves had poor TPI as measured with the reference ELISA method and 21% had excellent TPI.

#### 3.2.2. Diagnostic Test Characteristics

Diagnostic test characteristics for the identification of the category of TPI status are presented in Table 3. The quick test, either with EDTA or lithium heparin anticoagulants, performed better than the Brix refractometer in this study. Looking at test characteristics for the four categories, we observed that the category of poor TPI showed the best combination of precision and sensitivity for all methods, whereas the category of good TPI showed the worse results for all methods. The quick tests with EDTA vs. lithium heparin showed some differences in the results for the four categories although they had similar overall performances.

#### 3.2.3. Weighted Kappa (Agreement)

The weighted kappa statistics between the different tests in the current study is presented in Table 4. It may be seen that the agreement with ELISA was higher for the quick test performed with both of the anticoagulant types (weighted kappa = 0.80 and 0.78 for EDTA and lithium heparin, respectively, which are described as good agreement [38]) compared to refractometry which showed only moderate agreement with ELISA (weighted kappa = 0.57). The two quick tests showed good agreement between them (weighted kappa = 0.73).

## 4. Discussion

Estimating the quantity of IgG absorbed in blood serum following ingestion of colostrum by calves is essential to estimate the effectiveness of IgG passive transfer and subsequently take actions to prevent calf diseases. The main objective of this study was to assess the performance of the SmartStrips^TM^ quick immunochromatographic assay to quantify serum IgG concentration and assess TPI status in calves. Although some rapid qualitative immunoassays for assessment of TPI have been evaluated in the literature (e.g., [26,39,40]), we believe that the present study was the first to assess the performance of a rapid immunochromatographic assay for direct quantification (within the defined lower and upper detection limits) of bovine IgG concentration in blood serum.

### 4.1. Quantitative Analyses

In the current study, the average IgG concentration in calf serum measured by the reference method (i.e., ELISA, 13.6 mg/mL) was similar to that recently reported in other studies (e.g., 13 mg/mL [15], 13.3 mg/mL [41]), but lower than that reported by several other authors (e.g., 17.2 mg/mL [18], 21.3 mg/mL [29], 23.7 mg/mL [42]). Average values and standard deviations measured by the quick test (either with EDTA or lithium heparin) in this study were slightly lower than ELISA, most probably because of the existence of lower and upper detection limits for the quick test. The IgG values as measured by the reference method in the present study confirmed the generally low observed serum IgG concentrations in Belgian calves reported by Ronzoni (13.5 g/L) [31] and De Marchin and Theron (15.2 g/L) [32]. Aside from the animal and farm sampling strategy, reasons for discrepancy of IgG concentration between studies and countries are likely to be multifaceted, involving a number of the key factors like environmental, management, and feeding conditions [43]. Given the upper quantitative limit of 25 mg/mL of the quick test, the data used in the present study adequately cover the range of IgG values that could be measured by the immunochromatographic test. 

Pearson correlation coefficients showed that the quick test (either with EDTA or lithium heparin) results had a better linear relationship with IgG concentrations measured by the ELISA reference method compared to the digital Brix refractometer, even though the difference between both methods was limited in our study. In the literature, similar correlations between serum Brix scores and IgG concentration have been reported (e.g., r = 0.79 [29], r = 0.77 [15], r = 0.73 [27]), but higher correlations (as high as 0.93) have also been reported (e.g., [23]). It is important to note, however, that the reference method varied between studies. The wide range of correlation coefficients reported in the literature could be explained among others by variations in the source of serum samples, the instrument variation between refractometers and the non-Ig contents in serum affecting the indirect measures of the Brix refractometer [41]. The Bland-Altman plots showed that there was a negligible mean bias between the IgG concentration measured by the quick test (either with EDTA or lithium heparin) and the reference method. In contrast, the overall limits of agreement with ELISA were quite wide for both anticoagulants, but it should be noted that we observed a somewhat greater difference between measurements for higher IgG concentrations (i.e., larger errors), while for lower concentrations data were closer to each other and had better agreement (slight heteroscedasticity). Increased variability of the differences between measurements with rising concentrations can often be observed in analytical systems [44]. The reason for this remains speculative in the present study.

### 4.2. Qualitative Analyses

Categorization of dairy calves with successful TPI or failure of TPI was until recently based on serum IgG concentrations of ≥10 and <10 mg/mL, respectively [23,41,42]. However, in recent years, growing evidence has suggested that this single 10 mg/mL cut-off value needed to be reviewed based on calf morbidity and subsequent use of antibiotics, also to differentiate more easily calves that are the limit of failure of TPI or at lower risk [4,15]. Consequently, we defined four classes to categorize the level of TPI in calves, placing calves into well-proportioned categories with reasonably different risk groups. These categories were based on the new standard classification defined in the United States by Lombard et al. [4], but they were slightly adapted in accordance with the lower observed serum IgG values for Belgian calves and opinion of experts in veterinary medicine in order to have realistic goals. In accordance with the generally low observed serum IgG concentration values, the prevalence of calves with poor TPI (IgG < 10 mg/mL) was quite high in our study (33%). Compared with other studies in Belgium, it is consistent with what has been reported by Ronzoni for Belgian Blue calves (35%) [31] but slightly higher than the prevalence reported by De Marchin and Theron for Belgian Blue calves (25%) [32].

In the present study, based on diagnostic test characteristics, the quick test appeared to be more accurate in categorizing the TPI status than the digital Brix refractometer, irrespective of the blood anticoagulant type used. Our results were difficult to compare with the literature as we were, to our knowledge, the first to use new standards (not dichotomous) to categorize calves with different levels of TPI. Only the performances for the category of poor TPI could be directly compared with other studies using a dichotomous standard with cut-off value equivalent to 10 mg/mL IgG. The sensitivity of the Brix refractometer for this category (0.69) was lower than that reported in many other studies in the literature (e.g., 0.87 [27], 0.88 [18], 0.81 [45]). One reason might be the different Brix cut-off values in these studies that were usually optimized (e.g., using ROC curves) to obtain the best results. In contrast, in the present study, we used recommended Brix values equivalent to IgG concentrations to define the Brix thresholds, which is a more practical approach and similar to what would be done by practitioners in the field. Sensitivity of the quick test with EDTA anticoagulant for the category of poor TPI (0.83) was comparable to the sensitivity reported by McVicker et al. (0.89) [40] and by Elsohaby and Keefe (0.78) [26] for rapid qualitative immunoassays using calf serum. Sensitivity of the quick test with lithium heparin was higher (0.96) and comparable to the sensitivity reported by Dawes et al. (0.93) [39] for a commercially available rapid immunoassay, but the precision compared to the quick test with EDTA was lower (0.81 vs. 0.94 for lithium heparin vs. EDTA). Over the different methods, results for the categories of good and fair TPI of passive immunity were globally less satisfactory. In addition, the number of observations for the quick test with EDTA or lithium heparin were contrasted for these categories. This might be a consequence of the rather narrow ranges of IgG concentrations and %Brix for these categories combined with moderate instrument accuracy in this range of measures. In the sense that a major purpose of an on-farm test is to detect the presence and assess the proportion of calves with poor TPI status, the quick test seemed to be effective even if performances were less good in classifying higher levels of TPI. The overall level of agreement (weighted kappa) between the results derived from the quick test (irrespective of the anticoagulant type) and the reference method was good and higher than the level of agreement between the results from the Brix refractometer and the reference method. This confirms the better performances of the quick test compared to the digital Brix refractometer in this study.

### 4.3. Effect of the Anticoagulant Type

A secondary objective was to examine the effect of two different blood anticoagulants used during the blood collection on the performances of the quick test. Lithium heparin is the most widely used anticoagulant for clinical chemistry and its mechanism of action consists of enhancing the inhibitory activity of the plasma protein antithrombin against several serine proteases of the coagulation system, most importantly thrombin [46,47]. On the other hand, EDTA is widely used for hematology procedures and prevents clotting by binding calcium ions, preventing the coagulation proteins from using them [46]. The quantitative and qualitative results showed that there were no major differences between the results from both blood anticoagulant types used in the protocol of the quick test and both of them could be suitable. Indeed, the correlation between IgG concentrations measured by the quick test with EDTA vs. lithium heparin indicated a good linear relationship, the Bland-Altman plot between the measurements with the two anticoagulants showed an almost zero mean difference and reasonable limits of agreement, overall test characteristics were roughly equivalent and classification agreement was good. Even though the quick test with EDTA seemed to deliver slightly better results, this is difficult to confirm with certainty, and the little discrepancies between results from both anticoagulants might be due to errors or imprecisions of the assays or reading application and device. Repeatability study and increased number of samples would be helpful to further investigate the potential influence of the anticoagulant type.

### 4.4. Practical Considerations

In addition to the efficacy of the test at returning correct results, practical considerations may influence its use in particular circumstances. Many advantages exist of using the SmartStrips^TM^ quick assays to determine IgG concentration over currently available direct or indirect laboratory methods such as radial immunodiffusion (RID), ELISA, or enzyme gamma-glutamyl transferase measurement. For example, laboratory methods usually have long processing time (e.g., RID has an incubation time of 18 to 24 h [41]). The SmartStrips^TM^ quick test would take less than 15 min in total to obtain the results. A rapid method of measuring IgG is essential for quick identification of calves at risk of failure of TPI and to take early actions regarding for instance colostrum feeding practices and cow nutrition in the dry period. The affordable price and the simplicity of use of the SmartStrips^TM^ test compared to laboratory methods are also great advantages and will easily allow for on-farm applications. Advantages of the quick test compared to the commonly-used Brix refractometer are the direct measure of IgG concentrations and not Brix scores (i.e., values are more tangible and easier to use without conversion) and it does not require the use of a centrifuge to obtain serum as the test can be done on whole blood. One advantage of SmartStrips^TM^ test compared to other rapid immunoassays mentioned in the literature (e.g., [26,39,40]) is that they provide quantitative values of IgG concentration (within the defined detection limits) and not only a positive or negative result to assess if the cut-off point for adequate TPI was reached or not. This makes SmartStrips^TM^ tests more versatile. For example, tests with positive and negative values based on a cut-off point might be sufficient to establish if failure of TPI occurred, but this assumes the cut-off point used is appropriate for all particular circumstances and sufficient for all calves on all farms, which is not always the case. A semi-quantitative test such as SmartStrips^TM^ quick assay would allow easy adjustment of thresholds for TPI as well as the definition of several categories of TPI with more flexibility. Also, in the light of the actual need for prevention of treatment in calves, it is important to have several levels of values for the IgG transfer, in order to predict and measure the risk for pathology (to assess morbidity and welfare) and antibiotic treatment. 

From a practical point of view, the primary disadvantages of the SmartStrips^TM^ test are that immunochromatography is influenced by temperature (blood needs to be at ambient temperature), a smartphone is required to read the results, and most importantly the price of individual analyses can be high compared to other on-farm methods such as refractometry measurements. 

It should be noted that the Brix refractometer and the quick test were assessed in one geographical area (Belgium), thus the external validity of their performances may vary in different geographical conditions where serum IgG or total solids concentrations are different. Also, other factors such as the health status, hydration status, breed, age, or weight of the calves might influence the serum IgG concentration and test performances and could be investigated in future studies. Breed effect was not assessed per se in the present study as the number of observations for each breed was too limited and unbalanced for a fair comparison. Moreover, repeatability (i.e., variability in the measurements made on the same subject under identical conditions) and reproducibility (i.e., measurements made by different observers) of the quick test were not assessed in the present study and would be interesting to consider in further research.

## 5. Conclusions

In summary, result of the present study indicated that the SmartStrips^TM^ immunochromatographic assay would be a workable and appropriate on-farm method for quantification of serum IgG concentration and the categorization of TPI status in beef and dairy calves, particularly for identification of calves with poor TPI (serum IgG < 10 mg/mL). The quick test showed better performances compared to the Brix refractometer. Our results would suggest that EDTA or lithium heparin anticoagulants used in the blood collection tubes for the quick test would both be suitable. The convenience of being able to obtain rapid direct measurements of IgG with the quick test and the ease of use would provide producers and practitioners with a useful and affordable tool for calf management programs.

## Figures and Tables

**Table 1:** Transfer of passive immunity (TPI) categories.

TPI Category	IgG Category (mg/mL)	Equivalent Brix %
Excellent	≥20.0	≥9.0
Good	15.0–19.9	8.5–8.9
Fair	10.0–14.9	8.0–8.4
Poor	<10.0	<8.0

**Table 2:** Descriptive statistics of blood samples used in analyses.

Method	Measure	*n*	Mean	SD	Minimum	Maximum
ELISA	IgG (mg/mL)	97	13.6	7.5	1.3	37.1
Brix refractometer	Brix (%)	97	8.4	0.9	6.6	10.3
Quick test (SmartStrips^TM^) (EDTA)	IgG (mg/mL)	91	12.8	5.7	2.9	24.4
Quick test (SmartStrips^TM^) (lithium heparin)	IgG (mg/mL)	87	12.0	5.3	2.3	24.1

**Table 3:** Diagnostic test characteristics (4 categories of transfer of passive immunity (TPI)) for the SmartStrips^TM^ quick tests (with EDTA or lithium heparin anticoagulant) and the Brix refractometer compared to ELISA (*n* = 97 for all methods). See Table 1 for the definition of the categories.

Method	Macro F1 Score	Overall Accuracy	TPI Categories	Precision	Sensitivity	F1 Score
SmartStrips^TM^ (EDTA)	0.75	0.76	Excellent	0.70	1.00	0.82
			Good	0.86	0.50	0.63
			Fair	0.58	0.78	0.67
			Poor	0.94	0.83	0.88
SmartStrips^TM^ (lithium heparin)	0.72	0.76	Excellent	0.70	0.74	0.72
			Good	0.43	0.50	0.46
			Fair	0.90	0.72	0.80
			Poor	0.81	0.96	0.88
Brix refractometer	0.49	0.55	Excellent	0.70	0.50	0.58
			Good	0.14	0.14	0.14
			Fair	0.42	0.65	0.51
			Poor	0.75	0.69	0.72

**Table 4:** Matrix (lower part of the diagonal) displaying weighted kappa statistics between the different methods (ELISA, Brix refractometer, quick test (SmartStrips^TM^) with EDTA or lithium heparin anticoagulant).

	ELISA	Brix Refractometer	SmartStrips^TM^ (EDTA)	SmartStrips^TM^ (Lithium Heparin)
ELISA				
Brix refractometer	0.57			
SmartStrips^TM^ (EDTA)	0.80	0.50		
SmartStrips^TM^ (lithium heparin)	0.78	0.54	0.73	

## Data Availability

The data presented in this study are available on request from the corresponding author.
